# WHO-recommended levels of physical activity in relation to mammographic breast density, mammographic tumor appearance, and mode of detection of breast cancer

**DOI:** 10.1186/s13058-024-01889-4

**Published:** 2024-09-20

**Authors:** Öykü Boraka, Hanna Sartor, Li Sturesdotter, Per Hall, Signe Borgquist, Sophia Zackrisson, Ann H. Rosendahl

**Affiliations:** 1 Department of Clinical Sciences Lund, Oncology, Lund University and Skåne University Hospital, Lund, Sweden; 2 Department of Translational Medicine, Diagnostic Radiology, Lund University, Malmö, Sweden; 3 Department of Medical Epidemiology and Biostatics, Karolinska Institutet, Stockholm, Sweden; 4 Department of Oncology, Södersjukhuset, Stockholm, Sweden; 5 Department of Oncology, Aarhus University, Aarhus University Hospital, Aarhus, Denmark; 6 Department of Imaging and Physiology, Skåne University Hospital, Malmö, Sweden

**Keywords:** Breast cancer, Physical activity, Mammographic breast density, Mammographic tumor appearances, Mode of breast cancer detection

## Abstract

**Background:**

Despite known benefits of physical activity in reducing breast cancer risk, its impact on mammographic characteristics remain unclear and understudied. This study aimed to investigate associations between pre-diagnostic physical activity and mammographic features at breast cancer diagnosis, specifically mammographic breast density (MBD) and mammographic tumor appearance (MA), as well as mode of cancer detection (MoD).

**Methods:**

Physical activity levels from study baseline (1991–1996) and mammographic information from the time of invasive breast cancer diagnosis (1991–2014) of 1116 women enrolled in the Malmö Diet and Cancer Study cohort were used. Duration and intensity of physical activity were assessed according to metabolic equivalent of task hours (MET-h) per week, or World Health Organization (WHO) guideline recommendations. MBD was dichotomized into low-moderate or high, MA into spiculated or non-spiculated tumors, and MoD into clinical or screening detection. Associations were investigated through logistic regression analyses providing odds ratios (OR) with 95% confidence intervals (CI) in crude and multivariable-adjusted models.

**Results:**

In total, 32% of participants had high MBD at diagnosis, 37% had non-spiculated MA and 50% had clinical MoD. Overall, no association between physical activity and MBD was found with increasing MET-h/week or when comparing women who exceeded WHO guidelines to those subceeding recommendations (OR_adj_ 1.24, 95% CI 0.78–1.98). Likewise, no differences in MA or MoD were observed across categories of physical activity.

**Conclusions:**

No associations were observed between pre-diagnostic physical activity and MBD, MA, or MoD at breast cancer diagnosis. While physical activity is an established breast cancer prevention strategy, it does not appear to modify mammographic characteristics or screening detection.

**Supplementary Information:**

The online version contains supplementary material available at 10.1186/s13058-024-01889-4.

## Introduction

Physical activity is associated with a lower risk of at least 13 cancer types [1], including breast cancer [2, 3] and is recommended as a cancer preventive measure by establishments such as the World Health Organization (WHO) [4] and American Cancer Society [5]. A review of 5 cohort studies showed an inverse association between both moderate and vigorous intensity physical activity and breast cancer risk [6]. The intensity of physical activities are defined according to their metabolic equivalent of task (MET) values that are determined by the amount of oxygen consumed during an activity [7]. For health promoting benefits, the WHO recommends 150–300 min of moderate intensity (3–6 MET) activity per week (i.e. 7.5–30.0 MET-h/week), or 75–150 min of vigorous intensity (> 6 MET), or an equivalent combination of both moderate and vigorous activity. In line with the upper range of WHO guidelines, total physical activity corresponding to an energy expenditure equivalent to a minimum of one hour of daily walking (*≥* 28.5 MET-h/week) may reduce the risk of breast cancer [2]. While exercise reduces overall body fat and promotes a healthier body composition [8], the impact of physical activity on breast cancer determinants associated with mammographic features and cancer detection are poorly understood. Assessing these relationships have important clinical and public health implications that could improve personalized breast cancer risk assessments and preventive strategies, and aid mammographic interpretations.

Mammography is the primary imaging modality in breast cancer screening and diagnosis. Mammographic breast density (MBD) is a well-studied mammographic feature and an established breast cancer risk factor associated with up to 5-fold increased breast cancer risk [9]. Dense breast tissue is composed of the epithelial and fibrous (fibroglandular) tissue that appears white on a mammogram due to being radio opaque [10], which influences tumor visibility and can mask tumors on a mammogram, leading to increased risk of missing or delayed cancer diagnoses. MBD also impacts mammographic tumor appearance (MA) [11] which can provide valuable information on the aggressiveness of the tumor [12]. The mode of detection (MoD) is additionally important for breast cancer prognosis, whether the tumor is detected through the screening program or symptomatic through clinical manifestations, the latter of which is associated with worse prognosis [13].

The relationship between physical activity and MBD has been inconclusive due to heterogeneity in study designs, both with regards to physical activity definitions as well as MBD classifications being based on visual qualitative assessments or computer-aided quantitative measurements. A systematic review of 21 studies showed that the majority of the studies found no association between physical activity and MBD [14]. A study of the prospective Danish Diet, Cancer and Health cohort also found no association between baseline physical activity and MBD that was assessed qualitatively by radiologists and dichotomized as fatty and mixed/dense [15]. On the contrary, one study that measured MBD by using Volpara software showed that healthy women who engage in higher levels of physical activity had a higher percent MBD while having a lower absolute dense and nondense volume [16]. An earlier study of breast cancer patients that measured MBD by using Cumulus software also showed an association between post-diagnostic physical activity levels and dense area [17]. In addition, an intervention study that measured MBD by using Volpara documented a reduction in percent MBD upon 24 months of physical activity [18]. As for mammographic tumor appearance (MA) and mode of detection (MoD), there is no published research on the potential impact of physical activity on these parameters to the best of our knowledge.

The main objective of the present study was to investigate the associations between pre-diagnostic physical activity according to WHO-recommended and earlier reported breast cancer risk-reducing levels of physical activity and MBD, MA, and MoD at the time of breast cancer diagnosis. The secondary objective was to examine how different levels of moderate or vigorous activities relate to these mammographic features.

## Methods

### Study population

**Fig. 1:**
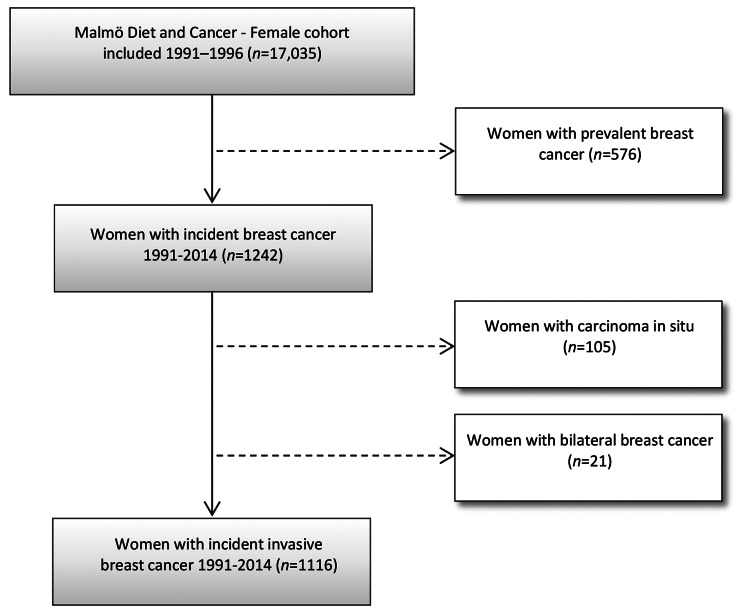
CONSORT flow diagram of study population

The Malmö Diet and Cancer Study (MDCS) is a large population-based prospective cohort which enrolled Malmö residents aged 44–74 years upon invitation from 1991 to 1996 [19]. At baseline, a comprehensive questionnaire reporting their socioeconomic position, reproductive and lifestyle factors, including physical activity was filled in, and anthropometric measurements were taken. The MDCS database is updated annually with information on vital status and incident breast cancer diagnoses that are retrieved through national registers: the Swedish Cause of Death Register, the Swedish Cancer Register, and the Regional Tumor Register for Southern Sweden. Among 17,035 female participants of the cohort, women who had prevalent breast cancer at baseline (*n* = 576), and women who were diagnosed with non-invasive breast cancer (carcinoma in situ) (*n* = 105) or bilateral breast cancer (*n* = 21), were excluded from the present study population, and the remaining 1116 women who were diagnosed with incident invasive breast cancer during the follow-up period 1991–2014 were included in the present study (Fig. 1). All study participants provided written informed consent. Ethical approvals for the MDCS (LU 51–90) and the present study (Dnr 652/2005, 2014/830) were obtained from the regional ethics committee in Lund.

### Physical activity assessment

Physical activity information was retrospectively retrieved for the year prior to study inclusion within the self-reported questionnaire at study baseline. Study participants recorded the number of minutes they engaged in 17 different types of physical activity weekly, in leisure time and transportation to and from work, during each of the four seasons of the previous year. The MDCS employed a physical activity questionnaire derived from a modified version of the Minnesota Leisure Time Physical Activity questionnaire [20]. This questionnaire’s validity was confirmed by comparing it to accelerometer data collected from a random subset of 369 male and female MDCS participants [21]. Physical activity information was used both as time spent (min/week) as well as computed as metabolic equivalent of task (MET) hours according to the 2011 Adult Compendium of Physical Activities [22]. MET-hours of physical activities were computed as described before [2].

Two approaches were employed to study the influence of total physical activity levels on mammographic features. The first approach was based on a previously reported breast cancer risk-reducing physical activity level, equivalent to a minimum of one hour daily walking, with total physical activity categorized into low or high levels using 28.5 MET-h/week as a cut-off [2]. The second approach was based on the WHO guidelines of recommended health promoting total physical activity levels [4]. The combined time spent on moderate and vigorous intensity activities was calculated based on the formula: 2 min of moderate (3–6 MET) activity = 1 min of vigorous (> 6 MET) activity. To obtain the equivalent combination of moderate and vigorous activity, the number of minutes the participants engaged in moderate intensity activities was weighted 0.5 and summed to the number of minutes spent on vigorous intensity activities, thereby the WHO guideline-subceeding/adhering/exceeding groups were created according to the WHO cut-offs for vigorous activity. Moderate and vigorous intensity activities were also categorized separately based on the number of minutes that are recommended by the WHO for moderate intensity < 150 (subceeding), 150–300 (adhering), ≥ 300 (exceeding) min/week; and vigorous intensity < 75 (subceeding), 75–150 (adhering), ≥ 150 (exceeding) min/week.

### Mammographic features

Mammographic images and reports were obtained from the time of breast cancer diagnosis as outlined elsewhere [23]. All screening mammograms were double read by two breast radiologists. Subsequent diagnostic imaging and mammograms from women with clinically detected cancers were read by one breast radiologist according to clinical routine. In case of missing information in the original mammography report, mammograms were re-examined by an experienced breast radiologist. 69 (6.2%) and 107 (9.6%) cases were missing for MBD and MA, respectively [23].

**Fig. 2:**
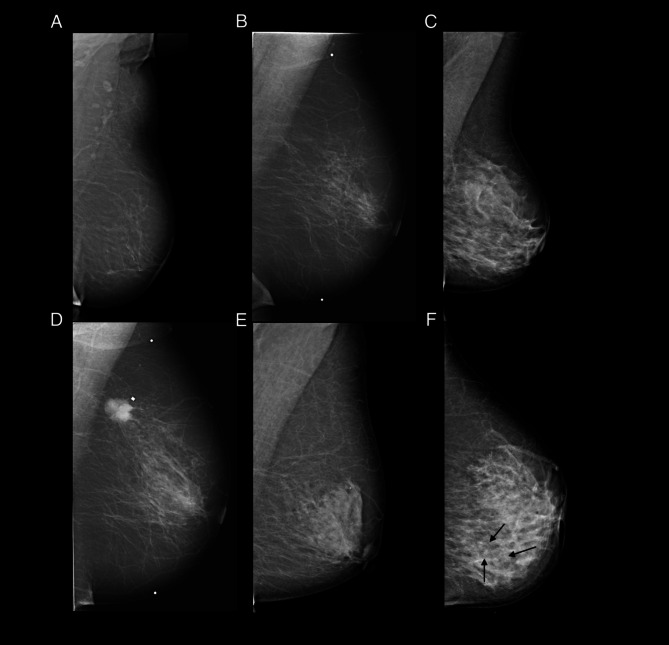
Mammographic images depicting different (**A–C**) mammographic breast densities and (**D–F**) tumor appearances. (**A**) Fat-involuted, (**B**) moderately dense, and (**C**) dense breast. (**D**) Spiculated mass, (**E**) distinct mass in the retro-mamillary, and (**F**) microcalcifications (shown with arrows)

Mammographic breast density (MBD) was qualitatively assessed at time point of imaging using both breasts and all views and divided into three groups at the Department of Breast Radiology in Malmö, Sweden, according to standard clinical praxis in Sweden: fat-involuted, moderately dense, and dense (Fig. 2A–C). MBD was subsequently dichotomized into low-moderate (fat-involuted or moderately dense) or high (dense). Fat-involuted corresponds to The Breast Imaging Reporting and Data System (BI-RADS) category 1, moderately dense corresponds to BI-RADS 2 and 3, and dense corresponds to BI-RADS 4 according to BI-RADS 4th edition [23]. Additionally, MBD categorization according to BI-RADS 5th edition [24], was available for the subset of patients diagnosed in 2008–2014 (*n* = 376) through a re-evaluation of the mammograms by HS [23]. Of these women, 218 (87%) classified as MBD low-moderate were correspondingly categorized as BI-RADS low (A + B), and 117 (94%) classified as MBD high were categorized as BI-RADS high (C + D) (Supplementary Table S1).

Mammographic tumor appearance (MA) information was retrieved from the original report and determined based on the most dominant appearance. Tumors were initially classified as: well-defined mass, partly ill-defined mass, ill-defined/diffuse mass, spiculated mass, comedo-type microcalcifications, non-specific calcifications, architectural distortion and asymmetrical density based on a previous work by Luck et al. [25] which were then grouped into 5 categories: distinct mass (well-defined and partly ill-defined), ill-defined mass, spiculated mass, calcifications (comedo-type and non-specific), and tissue abnormality (architectural distortion and asymmetrical density). For the present study, subsequent dichotomization into spiculated and non-spiculated (all other appearances) tumors was employed (Fig. 2D–F).

Mode of detection (MoD) was defined as clinically detected or screening detected. Interval cancers which are defined as a cancer diagnosed between two rounds of screening were included among the clinically detected cancers.

### Statistical analyses

The associations between physical activity and MBD (high vs. low), MA (non-spiculated vs. spiculated), and MoD (clinical vs. screening) were investigated by logistic regression analyses providing odds ratios (OR) with 95% confidence interval (CI) in both crude and adjusted models. The covariates included in the adjusted models were determined based on a priori known clinical associations with the mammographic outcomes, verified via univariable logistic regression within the present study cohort. Models for MBD were adjusted for age at diagnosis (continuous), menopausal status (premenopausal, perimenopausal, postmenopausal), body mass index (BMI at baseline, continuous), parity (0, 1, 2, 3, ≥ 4 children), oral contraceptive use (never, ever), current hormone replacement therapy (no, yes), and socioeconomic index (manual worker, nonmanual worker, employer/self-employed). Models for MA were adjusted for age at diagnosis (continuous), MBD (fat-involuted/moderately dense, dense), MoD (screening, clinical), age at first child (< 20, 21–25, 26–30, > 30 years). Models for MoD were adjusted for age at diagnosis (continuous), menopausal status (premenopausal, perimenopausal, postmenopausal), MBD (fat-involuted/moderately dense, dense), oral contraceptive use (never, ever), alcohol consumption (nothing last year, something last year, something last month), and socioeconomic index (manual worker, nonmanual worker, employer/self-employed). Sensitivity analyses were conducted for women with available BI-RADS data, and for pre- and peri-/postmenopausal women separately. Statistical analyses were performed using SPSS (version 29 for MAC, IBM).

## Results

### Distribution of baseline characteristics across groups of physical activity

Median time from study inclusion to breast cancer diagnosis was 10.9 years (interquartile range; IQR 6.1–15.5). Characteristics of the study population in relation to total physical activity levels are displayed in Table 1. Median age at baseline was 55 years (IQR 50–62) and at breast cancer diagnosis 66 years (IQR 61–73) and was overall similar between women reporting different levels of physical activity. Of the 1116 included participants, 1090 (98%) provided information on physical activity; 53% (*n* = 582) and 47% (*n* = 508) of whom reported a low vs. high MET-h/week, respectively, whereas women subceeding, adhering vs. exceeding WHO recommended physical activity levels were 11% (*n* = 122), 22% (*n* = 237), and 67% (*n* = 731), respectively. In total, 1076 women participated in moderate intensity activities, 18% (*n* = 198) of whom reported less than recommended 150 min/week, whereas 30% (*n* = 325) and 51% (*n* = 553) of women reported 150–300 (WHO-adhering) and ≥ 300 (WHO-exceeding) min/week physical activity, respectively. As for the 816 participants who undertook vigorous intensity activities, 67% (*n* = 548), 22% (*n* = 183) and 10% (*n* = 85) engaged in < 75 (WHO-subceeding), 75–150 (WHO-adhering) and ≥ 150 (WHO-exceeding) min/week physical activity, respectively.

**Table 1:** Characteristics of the study population in relation to total physical activity levels

		Physical activity levels based on				
		MET-h/week		WHO guidelines (activity min)		
	All women (*n*=1116)	Low (*n* = 582)	High (*n* = 508)	Subceed (*n* = 122)	Adhere (*n* = 237)	Exceed (*n* = 731)
**Patient characteristics**						
Age at baseline, median (IQR)	55 (50–62)	55 (50–62)	55 (49–62)	54 (50–60)	57 (51–63)	55 (50–62)
Age at diagnosis, median (IQR)	66 (61–73)	67 (62–73)	66 (60–73)	67 (62–72)	68 (62–73)	66 (61–73)
Menopausal status						
Premenopausal	337 (30)	165 (28)	162 (32)	38 (31)	59 (25)	230 (32)
Perimenopausal	93 (8)	54 (9)	37 (7)	13 (11)	18 (8)	60 (8)
Postmenopausal	686 (62)	363 (62)	309 (61)	71 (58)	160 (68)	441 (60)
BMI, kg/m^2^, median (IQR)	25 (23–28)	25 (23–28)	24 (23–27)	26 (23–28)	25 (23–29)	25 (23–28)
**Mammographic features**						
Mammographic breast density (MBD)						
Fat involuted	185 (17)	115 (21)	68 (15)	28 (24)	51 (23)	104 (15)
Moderately dense	505 (45)	259 (47)	233 (50)	54 (47)	110 (49)	328 (48)
Dense	357 (32)	178 (32)	169 (36)	34 (29)	63 (28)	250 (37)
Missing	69 (6)	30 (5)	38 (8)	6 (5)	13 (6)	49 (7)
BI-RADS						
A, almost entirely fatty	88 (23)	55 (27)	33 (20)	17 (32)	22 (29)	49 (21)
B, scattered areas of fibroglandular density	137 (36)	69 (34)	64 (39)	19 (35)	26 (34)	88 (37)
C, heterogeneously dense	121 (32)	68 (34)	48 (29)	14 (26)	26 (34)	76 (32)
D, extremely dense	30 (8)	11 (5)	19 (12)	4 (8)	3 (4)	23 (10)
Missing	740 (66)	379 (65)	344 (68)	68 (56)	160 (68)	495 (68)
Mammographic tumor appearance (MA)						
Spiculated	416 (37)	233 (44)	171 (38)	40 (36)	99 (46)	265 (40)
Distinct mass	266 (24)	140 (26)	119 (27)	27 (24)	54 (25)	178 (27)
Ill-defined mass	203 (18)	104 (20)	95 (21)	26 (23)	41 (19)	132 (20)
Calcifications	83 (7)	38 (7)	43 (10)	13 (12)	13 (6)	55 (8)
Tissue abnormality	41 (4)	19 (4)	21 (5)	5 (5)	7 (3)	28 (4)
Missing	107 (10)	48 (8)	59 (12)	11 (9)	23 (10)	73 (10)
Mode of cancer detection (MoD)						
Screening detection	555 (50)	301 (52)	240 (48)	65 (54)	118 (51)	358 (49)
Clinical detection	549 (50)	275 (48)	262 (52)	56 (46)	115 (49)	366 (51)
Missing	12 (1)	6 (1)	6 (1)	1 (1)	4 (2)	7 (1)
**Lifestyle and reproductive factors**						
Age at menarche						
≤12 years	256 (23)	137 (24)	110 (22)	34 (28)	53 (23)	160 (22)
13-14 years	581 (53)	303 (52)	267 (53)	70 (58)	120 (51)	380 (52)
>15 years	269 (24)	138 (24)	126 (25)	17 (14)	61 (26)	186 (26)
Missing	10 (1)	4 (1)	5 (1)	1 (1)	3 (1)	5 (1)
No. of births						
0	155 (14)	81 (14)	68 (14)	16 (13)	33 (14)	100 (14)
1	211 (19)	108 (19)	95 (19)	27 (23)	43 (19)	133 (19)
2	504 (46)	247 (43)	249 (50)	52 (43)	93 (40)	351 (49)
3	171 (16)	97 (17)	71 (14)	16 (13)	43 (19)	109 (15)
≥4	53 (5)	36 (6)	17 (3)	9 (8)	20 (9)	24 (3)
Missing	22 (2)	13 (2)	8 (2)	2 (2)	5 (2)	14 (2)
Age at first child birth						
≤20	179 (16)	98 (17)	78 (16)	24 (20)	39 (17)	113 (16)
21-25	391 (36)	207 (36)	177 (35)	38 (32)	84 (36)	262 (36)
26-30	258 (24)	116 (20)	134 (27)	22 (18)	49 (21)	179 (25)
>30	110 (10)	66 (12)	43 (9)	20 (17)	26 (11)	63 (9)
Nulliparous	155 (14)	81 (14)	68 (14)	16 (13)	33 (14)	100 (14)
Missing	23 (2)	14 (2)	8 (2)	2 (2)	6 (3)	14 (2)
Oral contraceptive use						
Never	513 (46)	264 (45)	233 (46)	51 (42)	114 (48)	332 (45)
Ever	602 (54)	318 (55)	275 (54)	71 (58)	123 (52)	399 (55)
Current use of HRT						
No	785 (71)	411 (71)	358 (71)	85 (70)	176 (75)	508 (70)
Yes	328 (29)	169 (29)	149 (29)	36 (30)	60 (25)	222 (30)
Alcohol use						
Nothing last year	94 (8)	51 (9)	37 (7)	13 (11)	26 (11)	49 (7)
Something last year	125 (11)	56 (10)	63 (12)	15 (12)	21 (9)	83 (11)
Something last month	895 (80)	474 (82)	408 (80)	94 (77)	189 (80)	599 (82)
Socieconomy						
Manual worker	374 (34)	196 (34)	169 (34)	44 (36)	90 (38)	231 (32)
Nonmanual worker	666 (60)	342 (59)	312 (62)	68 (56)	130 (55)	456 (63)
Employer/self-employed	66 (6)	40 (7)	24 (5)	9 (7)	15 (6)	40 (5)

Number and valid column % presented unless specified otherwise. Missing reported if > 1%, shown as total percentages. Low physical activity: <28.5 MET-h/week, high physical activity: ≥28.5 MET-h/week

Among the women reporting high MET-h/week and WHO guideline-exceeding levels, 36% and 37% had dense breasts, respectively, compared with 32% and 29% of women in the low MET-h/week and WHO guideline-subceeding groups, respectively. Overall, 15% of the women in the high MET-h/week and WHO guideline-exceeding groups had fat-involuted breasts compared with 21% and 24% of women in the low MET-h/week and WHO guideline-subceeding groups, respectively.

### Levels of physical activity in relation to different mammographic features

The median time spent (min/week) on different moderate or vigorous intensity activities according to mammographic features of MBD (low vs. high), spiculated vs. non-spiculated MA (spiculated vs. non-spiculated), or MoD (screening vs. clinical) are shown in Table 2. Of the participants, 1076 (99%) engaged in moderate intensity activities with a median duration of 315 (IQR 180–487) min/week, and 816 (75%) engaged in vigorous intensity activities 53 (IQR 28–90) min/week. In total, 1090 women engaged in 353 (IQR 218–555) min/week of total (moderate and/or vigorous) physical activity. The time spent on moderate/vigorous/total activity per week were similar between groups of MBD, MA, or MoD (Table 2).

**Table 2:** Physical activity levels according to mammographic features

		All women		MBD		MA		MoD	
	MET-value	Total activity		Low	High	Spiculated	Non-spiculated	Screening	Clinical
		*n* (%)	min/week	(*n* = 690)	(*n* = 357)	(*n* = 416)	(*n* = 593)	(*n* = 555)	(*n* = 549)
**Physical activities**									
Moderate	3.0–6.0	1076 (96)	315 (180–487)	300 (165–490)	329 (196–485)	298 (173–469)	330 (180–493)	300 (167–467)	330 (190–506)
Vigorous	> 6.0	816 (73)	53 (28–90)	52 (25–86)	55 (30–104)	50 (25–90)	51 (28–90)	51 (25–90)	56 (30–90)
All		1090 (100)	353 (218–555)	343 (210–535)	370 (240–568)	336 (218–534)	358 (219–560)	343 (214–532)	370 (225–568)
**Moderate intensity**									
Gardening	3.8	467 (42)	75 (35–143)	75 (45–147)	60 (30–135)	68 (30–125)	75 (38–149)	68 (30–121)	90 (45–151)
Cycling	4.0	700 (63)	108 (49–190)	100 (45–195)	120 (60–189)	100 (45–195)	120 (56–198)	100 (50–188)	120 (45–190)
Table tennis	4.0	3 (< 1)	40 (25–45)*	45 (45–45)	33 (25–40)*	25 (25–25)	43 (40–45)*	40 (25–45)	-
Walking	4.0	972 (87)	154 (76–270)	160 (75–288)	146 (83–238)	145 (75–237)	175 (83–300)	135 (71–254)	180 (90–295)
Golf	4.8	66 (6)	299 (131–480)	300 (136–488)	240 (122–409)	180 (90–480)	343 (141–491)	269 (136–446)	300 (130–480)
Digging	5.0	182 (16)	30 (15–63)	30 (15–67)	23 (8–60)	30 (14–45)	34 (15–71)	30 (14–60)	30 (15–69)
Badminton	5.5	21 (2)	30 (10–45)	19 (8–54)	23 (9–45)	23 (8–38)	30 (10–60)	15 (8–30)	34 (11–56)
Ballroom dancing	5.5	101 (9)	60 (30–161)	60 (24–143)	120 (60–180)	60 (30–120)	90 (24–171)	60 (23–120)	73 (30–180)
Folk dancing	5.5	36 (3)	90 (49–130)	90 (35–135)	90 (70–90)	100 (83–152)	90 (38–101)	90 (56–111)	90 (15–141)
Grass cutting	5.5	196 (18)	21 (11–30)	20 (11–30)	23 (11–30)	15 (11–30)	23 (15–45)	20 (10–30)	23 (11–30)
**Vigorous intensity**									
Soccer	7.0	1 (< 1)	15 (15–15)	-	15 (15–15)	-	-	-	15 (15–15)
Swimming	7.0	259 (23)	30 (15–56)	30 (15–50)	30 (13–53)	30 (15–52)	34 (15–60)	30 (15–53)	30 (15–60)
Keep-fit exercise/ aerobics	7.3	288 (26)	45 (34–75)	45 (34–75)	45 (30–68)	45 (34–75)	45 (30–71)	45 (30–83)	45 (34–69)
Tennis	7.3	12 (1)	54 (24–75)	49 (30–75)	68 (17–101)	38 (14–88)	60 (26–75)	54 (32–75)	45 (19–73)
Jogging	8.0	79 (7)	30 (15–69)	30 (23–75)	30 (15–68)	30 (17–60)	30 (15–75)	30 (16–74)	30 (15–64)
Walking stairs	8.0	640 (57)	30 (15–45)	30 (15–50)	28 (15–44)	30 (15–45)	30 (15–50)	29 (15–45)	30 (15–50)
Orienteering	9.0	2 (< 1)	53 (45–60)*	-	53 (45–60)*	-	60 (60–60)	45 (45–45)	60 (60–60)

Activity levels shown as median values of activity min/week (IQR) or (minimum-maximum)*

MET: metabolic equivalent of task, MBD: mammographic breast density, MA: mammographic tumor appearance, MoD: mode of detection

### Associations between total, moderate or vigorous physical activity and mammographic breast density

Overall, no clear associations were found between pre-diagnostic physical activity levels and MBD at breast cancer diagnosis (Table 3). Women engaging in high levels of total physical activity, corresponding to 1-hour daily walking or more (≥ 28.5 MET-h/week), had similar odds of having high MBD as women in the lower category of physical activity (OR_adj_ 1.05, 95%CI 0.75–1.40). Likewise, increasing levels of physical activity on a continuous scale showed no association with MBD.

Compared with women subceeding WHO-guidelines of total physical activity, women exceeding recommended levels showed a modest positive trend towards high MBD in the unadjusted model (OR_cru_ 1.40, 95% CI 0.91–2.14; *P*_trend_=0.023). However, this observation was weakened after accounting for age, BMI, reproductive and socioeconomic factors in the adjusted model (OR_adj_ 1.24, 95% CI 0.78–1.98; *P*_trend_=0.176). Adjusting for time between physical activity assessment at study inclusion and breast cancer diagnosis did not alter the results, nor if excluding BMI from the model. A similar observation was made when analyses were limited to moderate intensity activities (*P*_trend_=0.043). Further restricting analyses to vigorous intensity activity did not show an association with MBD for women adhering to or exceeding WHO-recommended levels, as compared with the subceeding group. The null association between total, moderate or vigorous intensity activity and MBD was further supported in sensitivity analyses in a subset of participants according to BI-RADS density categorization (Table 3).

**Table 3:** Associations between physical activity and mammographic breast density or BI-RADS density

	MBD			BI-RADS		
	*n* (%)	OR_cru_ (95% CI)^a^	OR_adj_ (95% CI)^b^	*n* (%)	OR_cru_ (95% CI)^a^	OR_adj_ (95% CI)^b^
**Physical activity**						
MET-hours/week						
Low (< 28.5)	552 (54)	1.00 (REF)	1.00 (REF)	203 (55)	1.00 (REF)	1.00 (REF)
High (≥ 28.5)	470 (46)	1.18 (0.91–1.53)	1.05 (0.79–1.40)	164 (45)	1.08 (0.71–1.65)	1.05 (0.66–1.65)
Continuous	1022 (100)	1.00 (1.00*-1.01)	1.00 (1.00*-1.01)	367 (100)	1.00 (1.00*-1.01)	1.00 (1.00*-1.01)
WHO guidelines						
Subceed	116 (11)	1.00 (REF)	1.00 (REF)	54 (15)	1.00 (REF)	1.00 (REF)
Adhere	224 (22)	0.94 (0.58–1.55)	0.96 (0.56–1.64)	77 (21)	1.21 (0.58–2.51)	1.15 (0.52–2.50)
Exceed	682 (67)	1.40 (0.91–2.14)	1.24 (0.78–1.98)	236 (64)	1.45 (0.78–2.69)	1.40 (0.72–2.72)
*p-*trend		**0.023**	0.176		0.215	0.269
Moderate intensity						
< 150 min	189 (19)	1.00 (REF)	1.00 (REF)	78 (21)	1.00 (REF)	1.00 (REF)
150–300 min	307 (30)	1.14 (0.75–1.65)	1.18 (0.77–1.81)	111 (31)	1.19 (0.65–2.18)	1.30 (0.68–2.49)
≥ 300 min	513 (51)	1.40 (0.98–2.00)	1.36 (0.92–2.01)	174 (48)	1.43 (0.82–2.49)	1.43 (0.80–2.58)
*p-*trend		**0.043**	0.110		0.191	0.245
Vigorous intensity						
< 75 min	510 (67)	1.00 (REF)	1.00 (REF)	192 (68)	1.00 (REF)	1.00 (REF)
75–150 min	173 (23)	1.17 (0.81–1.67)	1.20 (0.80–1.78)	65 (23)	0.93 (0.52–1.67)	1.01 (0.54–1.89)
≥ 150 min	78 (10)	1.42 (0.87–2.31)	1.28 (0.75–2.20)	27 (10)	1.28 (0.57–2.88)	1.25 (0.49–3.14)
*p-*trend		0.132	0.260		0.724	0.710

Odds ratios (OR) with 95% confidence intervals (CI) predicting the odds of having a high MBD or high BI-RADS (C + D) density in relation to increasing levels of physical activity. ^a^Crude model. ^b^Multivariable model adjusted for: age at diagnosis, menopausal status, BMI at baseline, parity, ever oral contraceptive use, current hormone replacement therapy, and socioeconomic index

MBD: mammographic breast density. BI-RADS: breast imaging-reporting and data system

*Lower 95% CI ≤ 0.998. Values in bold indicate *p* < 0.05

Given that breast density vary by age, associations between physical activity and MBD were additionally assessed according to menopausal status. As shown in Supplementary Table S2, increased odds of high MBD were observed among premenopausal women exceeding WHO-recommended levels of total or moderate intensity physical activity in the crude model, in line with that observed for all women. However, these observations were weakened and not sustained after multivariable adjustment. No clear associations between physical activity and MBD were observed among postmenopausal women.

### Pre-diagnostic physical activity in relation to mammographic tumor appearance and mode of cancer detection

With regards to MA, multivariable-adjusted analyses found no association between higher levels of physical activity according to MET-h/week or WHO-guideline recommendations and odds of non-spiculated vs. spiculated tumors (Table 4). Lastly in relation to MoD, there was no indication of a dose-dependent association between any of the physical activity assessments and odds of having a clinical detected vs. screening detected tumor (Table 4).

**Table 4:** Associations between physical activity and mammographic tumor appearance or mode of detection

	MA			MoD		
	*n* (%)	OR_cru_ (95% CI)^a^	OR_adj_ (95% CI)^b^	*n* (%)	OR_cru_ (95% CI)^a^	OR_adj_ (95% CI)^b^
**Physical activity**						
MET-hours/week						
Low (< 28.5)	534 (54)	1.00 (REF)	1.00 (REF)	576 (53)	1.00 (REF)	1.00 (REF)
High (≥ 28.5)	449 (46)	1.26 (0.97–1.63)	1.24 (0.95–1.61)	502 (47)	1.20 (0.94–1.52)	1.25 (0.96–1.63)
Continuous	983 (100)	1.00 (1.00*-1.01)	1.00 (1.00*-1.01)	1078 (100)	1.00 (1.00*-1.01)	1.00 (1.00*-1.01)
WHO guidelines						
Subceed	111 (11)	1.00 (REF)	1.00 (REF)	121 (11)	1.00 (REF)	1.00 (REF)
Adhere	214 (22)	0.65 (0.41–1.05)	0.62 (0.38–1.02)	233 (22)	1.13 (0.73–1.76)	1.20 (0.74–1.94)
Exceed	658 (67)	0.84 (0.56–1.27)	0.78 (0.50–1.20)	724 (67)	1.19 (0.81–1.75)	1.32 (0.86–2.00)
*p-*trend		0.986	0.733		0.393	0.201
Moderate intensity						
< 150 min	178 (18)	1.00 (REF)	1.00 (REF)	196 (18)	1.00 (REF)	1.00 (REF)
150–300 min	295 (30)	0.97 (0.67–1.41)	0.96 (0.65–1.42)	321 (30)	1.17 (0.82–1.66)	1.17 (0.79–1.71)
≥ 300 min	497 (51)	1.19 (0.84–1.69)	1.17 (0.82–1.68)	547 (51)	1.29 (0.93–1.79)	1.26 (0.89–1.80)
*p-*trend		0.206	0.267		0.126	0.205
Vigorous intensity						
< 75 min	504 (68)	1.00 (REF)	1.00 (REF)	542 (67)	1.00 (REF)	1.00 (REF)
75–150 min	163 (22)	0.91 (0.64–1.30)	0.89 (0.62–1.29)	182 (23)	1.26 (0.90–1.76)	1.28 (0.88–1.85)
≥ 150 min	75 (10)	1.21 (0.73–2.00)	1.17 (0.69–1.97)	84 (10)	1.07 (0.68–1.70)	1.04 (0.62–1.72)
*p-*trend		0.722	0.848		0.392	0.479

Odds ratios (OR) with 95% confidence intervals (CI) predicting the odds of having a non-spiculated MA or clinical MoD in relation to increasing levels of physical activity. ^a^Crude model. ^b^Multivariable model adjusted for (MA): age at diagnosis, mammographic breast density, mode of cancer detection, age at first child; and for (MoD): age at diagnosis, menopausal status, mammographic breast density, ever oral contraceptive use, alcohol consumption, and socioeconomic index

MA: mammographic tumor appearance. MoD: mode of cancer detection

*Lower 95% CI ≤ 0.998

## Discussion

Our study has shown no clear associations between pre-diagnostic physical activity according to WHO guidelines or breast cancer-reducing levels and MBD at breast cancer diagnosis. Modest positive trends were observed between total and moderate intensity physical activity and MBD in unadjusted models, with effects primarily seen in premenopausal women, but the associations were weakened after adjustments for age, BMI, reproductive and socioeconomic factors. Furthermore, to the best of our knowledge, this is the first study to examine total, moderate or vigorous intensity physical activity in relation to MA and MoD. This study found no indications of physical activity being associated with spiculated or non-spiculated tumor appearance, or whether a breast cancer is detected through screening or clinically.

Research around physical activity and MBD have yielded conflicting results due to the variations in physical activity measurements and cut-offs as well as variations in the MBD assessments (qualitative vs. quantitative) and study designs. In line with our present findings, a Danish study in women without breast cancer showed no association between baseline physical activity and MBD that was qualitatively appointed by two radiologists in a multivariable adjusted (including BMI) logistic regression model [15]. Other studies that made use of percent MBD also found no association [26–31]. One study on postmenopausal women which used both absolute (cm^2^) and percent dense area measurements that were gathered during screening and computed by using Cumulus software also reported no association in a multivariable adjusted (including BMI) logistic regression model [32]. This study, however, categorized physical activity into groups (never, < 1 h/week, 1–2 hours/week, > 2 hours/week) that are below the WHO recommendations or the breast cancer risk-reducing level of physical activity we previously reported (1 h of walking/day). On the other hand, another study that computed volumetric MBD by using Volpara on raw screening mammograms found an inverse association between physical activity and both absolute dense and absolute non-dense volume (cm^3^), while showing a positive association with percent dense volume [16]. Physical activity is well-documented as a breast cancer preventive measure [4, 5] that may also be associated with better survival [33]. A recent prospective cohort study among physically inactive adults showed a reduced risk for total cancer and a more pronounced reduction in physical inactivity-related cancer with vigorous intermittent lifestyle physical activity (VILPA) defined as brief and sporadic (e.g., up to 1–2 minutes) bouts of vigorous physical activity, such as bursts of very fast walking or stair climbing, on a daily basis [34]. A Mendelian randomization study provided further support of a causal association between greater overall and vigorous physical activity and lower breast cancer risk [35]. Although, how physical activity mechanistically exerts this influence on breast cancer has not completely been understood, physical activity has been shown to modulate estrogen [36] and insulin signaling [37]. Its relationship with MBD is rather complex because physical activity may influence both fibroglandular and adipose tissues as also documented with epidemiological studies [16, 38] and high MBD is associated with low BMI [39]. We only observed two modest trends between high MBD and increasing levels of physical activity in the WHO guidelines total (*P*_trend_=0.023) and moderate activity (*P*_trend_ =0.043) groups in the unadjusted model that appeared mainly driven by premenopausal participants. However, the trends were attenuated in the multivariable-adjusted model, and not observed among postmenopausal women. The found trends in the unadjusted model could be stemming from the adipose mass-reducing effect of physical activity [40]. Also, the inhibitory effects of physical activity on estrogen signaling and fibroglandular tissue growth [41] probably were not captivated in our largely postmenopausal cohort.

To the best of our knowledge, this is the first study to investigate the relationship between physical activity and MA and MoD. Spiculated lesions indicative of malignant tumors, were found to be associated with favorable tumor characteristics [12] and a high survival rate [42–44], whereas the survival rate for women with certain tumor-associated calcifications was reported to be low [43]. Another study has shown no clear association between MA and breast cancer-specific survival [23]. Clinically, the present findings indicating that physical activity is not associated with MA or MoD would suggest that while physical activity is an established preventive strategy for breast cancer, it does not need to be factored into the evaluation of mammographic features or the detection process.

Our study has several strengths. MDCS cohort is a large and well-characterized prospective cohort including 1116 breast cancer diagnoses with detailed information on covariates allowing for relevant adjustments, and extensive physical activity data. It is worth to mention the availability of information on MA and MoD – there are no prior studies that have investigated these features in relation to physical activity. Our study has several limitations. One key limitation was that the physical activity questionnaire was self-reported at the study inclusion for the year prior to enrollment which means that the physical activity information may be prone to recall bias and further not represent their actual physical activity levels over the years until breast cancer diagnosis. Another limitation is the unavailability of mammograms from study baseline for the entire MDCS cohort which otherwise could enable analyzing the relationship between physical activity and MBD change in healthy women developing breast cancer. Furthermore, qualitative assessment by radiologists, as compared with automated quantitative methods, do not provide precise percent density measurement. Also, qualitative assessment of mammograms prevented from computing, thus, investigating physical activity in relation to absolute density in terms of fibroglandular area or volume.

## Conclusions

In conclusion, no association between pre-diagnostic physical activity and MBD, MA, or MoD at time of breast cancer diagnosis was found in our cohort. The lack of association was observed both in women with breast cancer risk-reducing levels of physical activity and women with WHO guideline-exceeding levels of physical activity. From a population health perspective, confirming the observed null association in independent prospective study populations would reinforce the benefits of physical activity for breast cancer prevention, without influence on mammographic characteristics or screening detection.

## Electronic supplementary material

Below is the link to the electronic supplementary material.

Supplementary Material 1

## Data Availability

The datasets generated and/or analyzed during the current study are not publicly available due to legal, ethical and privacy restrictions to protect patient confidentiality. Request to access the dataset should be directed to the corresponding author (AR) and the steering committee of the MDCS study (https://www.malmo-kohorter.lu.se/malmo-cohorts).

## Abbreviations

BI-RADS: Breast Imaging Reporting and Data System
BMI: Body Mass Index
CI: Confidence Interval
IQR: Interquartile Range
MA: Mammographic Tumor Appearance
MBD: Mammographic Breast Density
MDCS: Malmö Diet and Cancer Study
MET: Metabolic Equivalent of Task
MoD: Mode of Cancer Detection
OR: Odds Ratio
WHO: World Health Organization
