# Supplementary material for: WHO-recommended levels of physical activity in relation to mammographic breast density, mammographic tumor appearance, and mode of detection of breast cancer
Source: Breast Cancer Res. 2024 Sep 20;26:136. doi: 10.1186/s13058-024-01889-4 (PMC11414304; doi:10.1186/s13058-024-01889-4)
Supplement: Supplementary file 1 — Supplementary Material 1 [file 13058_2024_1889_MOESM1_ESM.pdf]

Supplementary Information

WHO-recommended levels of physical activity in relation to mammographic breast density, mammographic tumor appearance, and mode of detection of breast cancer

Öykü Boraka[1], Hanna Sartor[2], Li Sturesdotter[2,6], Per Hall[3,4], Signe Borgquist[1,5], Sophia Zackrisson[2,6], Ann H Rosendahl[1*]

Supplementary Table S1. Density classification concordance across categories of MBD and BI-RADS density in the subset of women with available BI-RADS data in the MDCS cohort

	MBD		
	Fat involuted	Modertely dense	Dense
**BI-RADS**			
A, almost entirely fatty	79 (93)	9 (5)	0 (0)
B, scattered areas of fibroglandular density	6 (7)	124 (74)	7 (6)
C, heterogeneously dense	0 (0)	34 (20)	87 (70)
D, extremely dense	0 (0)	0 (0)	30 (24)

Frequency distribution with total number of women and valid column percentages in parenthesis, n (%).

Supplementary Table S2. Associations between physical activity and mammographic breast density by menopausal status

	Premenopausal			Postmenopausal		
	***n* (%)**	**OR_{cru} (95% CI)[a]**	**OR_{adj} (95% CI)[b]**	**n (%)**	**OR_{cru} (95% CI)[a]**	**OR_{adj} (95% CI)[b]**
**PHYSICAL ACTIVITY**						
MET-hours/week						
Low (<28.5)	160 (51)	1.00 (REF)	1.00 (REF)	392 (55)	1.00 (REF)	1.00 (REF)
High (≥28.5)	152 (49)	1.25 (0.80–1.96)	1.03 (0.63–1.69)	318 (45)	1.12 (0.81–1.54)	1.04 (0.73–1.48)
Continuous	312 (100)	1.00 (0.99-1.01)	1.00 (0.99-1.01)	710 (100)	1.00 (1.00*-1.01)	1.00 (1.00*-1.01)
WHO guidelines						
Subceed	37 (12)	1.00 (REF)	1.00 (REF)	79 (11)	1.00 (REF)	1.00 (REF)
Adhere	56 (18)	1.88 (0.77–4.63)	1.93 (0.73–5.13)	168 (24)	0.72 (0.39–1.30)	0.70 (0.36–1.35)
Exceed	219 (70)	**2.27 (1.05–4.91)**	1.82 (0.79–4.21)	463 (65)	1.10 (0.67–1.84)	1.01 (0.57–1.78)
p-trend		**0.042**	0.267		0.222	0.458
Moderate intensity						
<150 min	59 (19)	1.00 (REF)	1.00 (REF)	130 (19)	1.00 (REF)	1.00 (REF)
150-300 min	92 (30)	1.48 (0.75–2.94)	1.57 (0.75–3.31)	215 (31)	0.97 (0.60–1.58)	1.05 (0.62–1.78)
≥300 min	158 (51)	**1.90 (1.01–3.57)**	1.63 (0.83–3.20)	355 (51)	1.20 (0.78–1.87)	1.24 (0.76–2.00)
p-trend		**0.043**	0.204		0.291	0.325
Vigorous intensity						
<75 min	169 (68)	1.00 (REF)	1.00 (REF)	341 (67)	1.00 (REF)	1.00 (REF)
75-150 min	47 (19)	1.69 (0.88–3.23)	1.56 (0.76–3.18)	126 (25)	1.04 (0.67–1.63)	0.98 (0.60–1.60)
≥150 min	34 (14)	1.32 (0.63–2.77)	1.43 (0.65–3.19)	44 (9)	1.38 (0.71–2.66)	1.17 (0.55–2.49)
p-trend		0.227	0.230		0.411	0.791

Odds ratios (OR) with 95% confidence intervals (CI) predicting the odds of having a high MBD in relation to increasing levels of physical activity and menopausal status. [a]Crude model. [b]Multivariable model adjusted for (premenopausal women): age at diagnosis, BMI at baseline, parity, ever oral contraceptive use, and socioeconomic index; and for (postmenopausal women): age at diagnosis, BMI at baseline, parity, ever oral contraceptive use, current hormone replacement therapy, and socioeconomic index.
*Lower 95% CI ≤0.997. Values in bold indicate $p<0.05$.
